# Understanding Brain-Skeletal Muscle Crosstalk Impacting Metabolism and Movement

**DOI:** 10.15190/d.2022.3

**Published:** 2022-03-01

**Authors:** Bhanu P. Jena, Lars Larsson, Domenico L. Gatti, Ionita Ghiran, Won Jin Cho

**Affiliations:** ^1^Department of Physiology, School of Medicine, Wayne State University, Detroit, MI, USA; ^2^NanoBioScience Institute, Wayne State University, Detroit, MI, USA; ^3^Center for Molecular Medicine and Genetics, School of Medicine, Wayne State University, Detroit, MI, USA; ^4^Viron Molecular Medicine Institute, Boston, MA, USA; ^5^Department of Physiology and Pharmacology, Karolinska Institutet, SE-171 77 Stockholm, Sweden; ^6^Biochemistry, Microbiology and Immunology, School of Medicine, Wayne State University, Detroit, MI, USA; ^7^Division of Allergy and Inflammation, Beth Israel Deaconess Medical Center, Harvard Medical School, Boston, MA, USA

**Keywords:** Metabolism, Movement, Brain, Skeletal Muscle.

## Abstract

Metabolism and movement, among the critical determinants in the survival and success of an organism, are tightly regulated by the brain and skeletal muscle. At the cellular level, mitochondria -that powers life, and myosin - the molecular motor of the cell, have both evolved to serve this purpose. Although independently, the skeletal muscle and brain have been intensively investigated for over a century, their coordinated involvement in metabolism and movement remains poorly understood. Therefore, a fundamental understanding of the coordinated involvement of the brain and skeletal muscle in metabolism and movement holds great promise in providing a window to a wide range of life processes and in the development of tools and approaches in disease detection and therapy. Recent developments in new tools, technologies and approaches, and advances in computing power and machine learning, provides for the first time the opportunity to establish a new field of study, the ‘Science and Engineering of Metabolism and Movement’. This new field of study could provide substantial new insights and breakthrough into how metabolism and movement is governed at the systems level in an organism. The design and approach to accomplish this objective is briefly discussed in this article.

SUMMARY

*1. Introduction*

*2. Design and Approach: Understanding Metabolism and Movement*

- *2.1 Focus 1: How brain and skeletal muscles communicate to regulate metabolism*

- *2.2 Focus 2: How skeletal muscle adapts to brain inputs and how exercise influences brain neural network*

- *2.3 Focus 3: Computational machine learning approach to capture key elements in brain-muscle cross-talk, function, and regulation* *3. *

*3. Conclusion*

## 1. Introduction

Metabolism and movement, among the critical determinants in the survival and success of an organism, are tightly regulated by the brain and skeletal muscle. While the skeletal muscle and brain have independently been investigated for over a century, their coordinated involvement in metabolism and movement remains poorly understood. For example, while skeletal muscle and the impact of exercise on body metabolism is well documented and studies report the tight correlation between metabolism and brain function^1^, there exists a brain-muscle cross-talk regulating metabolism and movement homeostasis^2,3^, which is little understood. Similarly, at the cellular level, the mitochondria is tasked with the synthesis of ATP, the molecule that fuels life processes, including the molecular motor myosin for movement. Although the mechanism of ATP synthesis by the mitochondria and it’s use by myosin for movement has greatly advanced, the cross-talk between mitochondria and myosin remains poorly understood. Recent studies report the contribution of myosin to mitochondrial DNA maintenance^4^. Clearly, there is a void in our knowledge on the crosstalk between metabolism and movement, and therefore requires intense research, with the outreaching goal to unravel the coordinated workings of the brain and skeletal muscle in the regulation of metabolism and movement and gain a systems-level molecular understanding of the cross-talk between mitochondria and myosin. To accomplish this objective, one could use *Drosophila*, and human primary neuron and muscle-on-a-chip, that will serve as the primary model systems. Noninvasive studies on humans could be conducted utilizing Magnetoencephalography (MEG),Positron Emission Tomography (PET) and functional Magnetic Resonance Imaging (fMRI) to determine brain and muscle activity and metabolism. In view of this, the following design and approach to progress our understanding of metabolism and movement at the systems level, is discussed in this article.

## 2. Design and Approach: Understanding Metabolism and Movement

The immediate research needed in gaining a systems level understanding of metabolism and movement will require a concentrated effort on the following three primary areas. *Focus 1: How do brain and skeletal muscles communicate to regulate metabolism;(a)* How different afferent and efferent inputs from the brain impacts skeletal muscle efficiency, motility and metabolism. *(b)* How exercise-induced exosomes released from skeletal muscles impact brain metabolism. *Focus 2: How does skeletal muscle adapt to brain inputs and vice versa; (a)*How visual cues related to exercise, a psychobiological model of endurance performance, impact skeletal muscle metabolism and function. *(b)*How exercise and sedentary states impact mitochondria and myosin remodeling in both the skeletal muscle and the brain. Focus 3: Computational approaches of machine learning could be used to capture key elements in brain-muscle cross-talk, function and regulation of metabolism and movement.

### 2.1 Focus 1: How brain and skeletal muscles communicate to regulate metabolism

#### (1a) How different afferent and efferent inputs from the brain impacts skeletal muscles will be determined

During exercise afferents from skeletal muscle become active due to insufficient O_2_ delivery. These afferents travel to the brain and elicit activation of autonomic nervous system efferents. Insufficient cardiac output results in these afferents to become hyperactive and are responsible for excessive increases in sympathetic output causing profound peripheral vasoconstriction. This vasoconstriction includes the heart and even the under-perfused skeletal muscle which thereby elicits a positive-feedback vicious cycle scenario acting as an effective amplifier of sympathetic activity. Noninvasive MEG,PET and fMRI could be used to determine brain and skeletal muscle activity and metabolism in animals. Skeletal muscle biopsies from animals from these studies will serve in determining at the cellular and molecular level, the remodeling of mitochondria and myosin, at the morphological, compositional and functional level could be examined using Electron Microscopy, STED, STORM, Expansion Microscopy, Atomic Force Microscopy, TIRF Microscopy, Electron transport chain proteins, mitochondrial biochemistry,myosin-actin motility assays,Mass Spectrometry Proteomics andLipidomics.

#### **(1b)** How exercise-induced exosomes released from skeletal muscles impact brain metabolism

**Figure 1:**
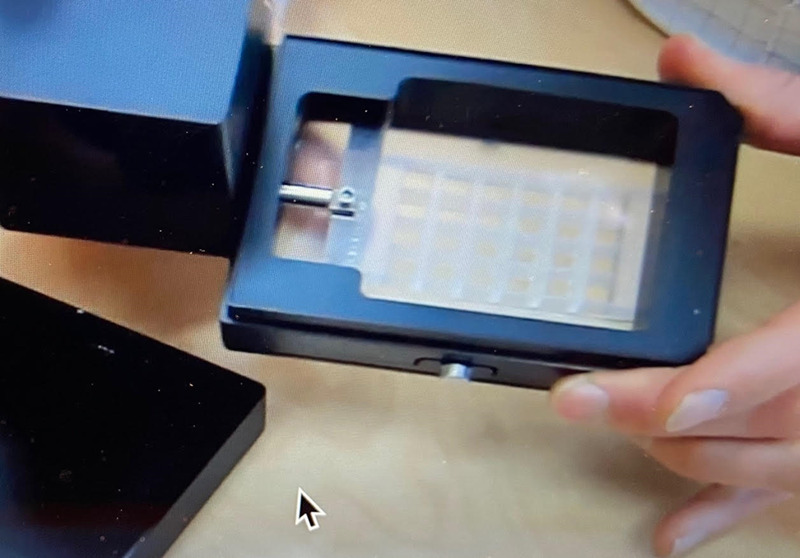
Multimodal tension device with stretch capacity mimicking both in tension and frequency, exercising native human skeletal muscles on a micropatterned 3D stretchable platform. The 3D stretchable platform is a single unit with sixteen culture wells each measuring 8mm x 8mm. The entire unit can be placed in a cell culture incubator in the laboratory and remotely controlled, and the cultured human skeletal muscle cells can be exposed to different physiochemical and biomechanical environments, to understand inactivity-induced human skeletal muscle myopathy. Silicone plate cell culture wells with a 0.25mm thick transparent bottom membrane (CellScale, Ontario, CA) on which our microphysiological 3D stretchable Poly-L-lysine-polydopamine (PDA)-coated parafilm membranes with micropatterned 10 µm groove spacings are attached. This setup allows uniaxial stretching and imaging in real time^7^.

Exosomes, the 40-120 nm membrane-bound vesicles released from cells are the natural carriers of miRNA, non-coding RNA and proteins, actively contributing to cell-cell communication^5^. Blood samples from sedentary and exercised animals used in studies outlined in **1a** will be used to isolate and determine circulating exosomes for characterization. These studies could be further complemented and validated using the ‘muscle-on-a-chip’ micro-physiological stretchable platform developed in the Jena laboratory (Figure 1), to obtained exosomes released from skeletal muscle cells in culture, following various regimes of stretched (exercised) and sedentary states and study their impact on the metabolism of brain neurons in culture. The Jena laboratory has optimized a stretchable micropatterned 3D skeletal muscle cell platform for use in studies that recapitulates organized and parallel growth of muscle fibers and cells^6^expressing key myogenic and mitochondrial proteins^7^. The proteome, lipidome and miRNA of the isolated exosomes, and the compositional remodeling of neurons as a result of exposure to skeletal muscle-derived exosomes from exercised and sedentary states, could be assessed. Similarly, the structural remodeling of the mitochondria in the cultured brain neurons subjected to the exosomes, could also be assessed using Differential Expansion Microscopy (DiExM)^8^. Complementing these studies, the effect of exosomes derived from the blood in exercised animals on brain metabolism of sedentary animals could be studied using MEG,PET and fMRI to provide valuable information on exercise-induced exosomes from skeletal muscles on brain function and metabolism.In 2015, as opposed to the invention of an imaging tool, the substrate was enlarged to enable nanoscale imaging using an ordinary diffraction limited light microscope^9^. In this simple yet novel approach termed expansion microscopy (ExM)^9^, a hydration-competent polymer (sodium polyacrylate) is used to physically expand biological specimens to be imaged at nm scale, using a light microscope^9-14^. ExM holds great promise for a fundamental understanding of the cell at the nanometer scale using a simple diffraction limited light microscope, making the approach cost effective and rapid (compared to immune electron microscopy or super resolution microscopy). To establish a correction factor that could be applied to the DiExM images, the Jena lab. in collaboration, has developed a Matlab-based interactive machine learning application for the analysis of both expanded and unexpanded images, capable of recognizing fine cellular changes affecting the size, shape and distribution of organelles and/or macromolecules. This machine learning approach is being further developed, to predict expanded status of cells and of any remodeling at the nanometer scale in the skeletal muscle and brain tissue^8^.

### 2.2 Focus 2: How skeletal muscle adapts to brain inputs and how exercise influences brain neural network

#### (2a) How visual cues related to exercise, a psychobiological model of endurance performance, impact skeletal muscle metabolism and function

A subset of neurons whose activity is necessary and sufficient to drive exercise adaptations even in sedentary animals have been identified. It has been demonstrated that octopamine (the invertebrate analog of norepinephrine) secretion during exercise mediates the effect of these neurons on exercise adaptation. It has been found that transient stimulation of neurons that secrete octopamine is sufficient to provide metabolic and performance benefits of exercise to sedentary animals. A follow up on these observations to identify downstream genetic mediators of these effects and understand the role of neural plasticity in the brain as an exercise mediator is therefore warranted. These efforts will advance our understanding of the mechanism at the molecular level and in its future application in the benefits of neural stimulation in humans. Furthermore, the relationship between visual cues and its impact on the skeletal muscle and brain metabolism should be further explored using MEG,EEG,PET and fMRI. Neuroimaging techniques such as MEG, EEG, PET and fMRI in understanding how the brain processes language, hearing, vision, sensory, memory and metabolic functions, will be able to assess metabolic changes in both skeletal muscles and the brain in these studies. We expect these combined efforts to simultaneously inform our understanding of exercise physiology, while progressing our understanding of the mechanisms by which animals and humans adapt to changing energy requirements.

#### (2b) How exercise and sedentary states impact mitochondria and myosin remodeling in both the skeletal muscle and the brain

Space flight and inactivity has been reported to induce a programed shift from slow to fast type II fibers in human skeletal muscles^15-18^. However, the molecular mechanism of this process remains unclear. Since physical activity in humans and animal models is known to upregulate the expression of the transcriptional coactivator PGC-1α involved in mitochondrial biogenesis and found to rescue animal models of myopathy^19^, the impact of exercise on the increased and decreased expression of PGC-1α on myosin and mitochondria remodeling in both the skeletal muscle and brain, could be studied using the fruit fly *Drosophila *and skeletal muscle and brain neuron cultures on the 3D microphysiological platform^7^.Similar to the disuse-induced muscle wasting in experimental rat model, muscle wasting as a result of disuse is seen in humans^20-23^ and the fruit fly^24-26^^24-26^^24-26^. To mimic muscle disuse in the fly, the movement of flies is restricted by limiting availability of space using a foam plug. The disuse assay system is suitable for both large-scale fly genetics and for longitudinal studies to follow individual changes in physiology of the fly under various conditions and during aging. Similarly, an earlier study in the Jena laboratory^27^ showed that the *Drosophila* homolog of the vertebrate exercise response gene PGC-1α *spargel* (srl) is sufficient to induce exercise-dependent phenotypes. Reduction of srl expression levels acutely compromises negative geotaxis ability and reduces exercise-induced improvement in both negative geotaxis and time to exhaustion. To test the hypothesis that overexpression of *spargel* would potentiate efficiency of the fly muscle in the use of ATP, extensive studies have been conducted in the Jena laboratory^19^ utilized cadmium telluride quantum dots (CdTe QDs) as nanothermometers. The CdTe QD’s in direct association with isolated skeletal muscles obtained from wild type *Drosophila melanogaster* outcrossed genetic background control flies (*y1w1UAS-srl)* and flies with muscle-specific *spargel *overexpression (*mef2>UAS-srl)*, demonstrate *spargel *overexpression to indeed potentiates muscle efficiency in the fly. Remodeling of the neural network in the fly brain, and of myosin and mitochondria in the fly brain and flight muscle could be assessed using DiExM, solution x-ray^27^ and neutron scattering and cryo-electron microscopy. The fly brain is composed of nearly 100,000 neurons^28^. Large dimension, high-resolution imaging is important for neural circuit visualization as neurons have both long- and short-range patterns, from axons and dendrites to synapses at nerve terminals. Although EM is the conventional approach for nanoscale imaging, tomograms are time consuming, tedious and expensive, and the retrieval of structural information segmented from high-density images within large volume datasets continues to remain challenging. This limitation could be overcome by using fluorescent probes to localize synapse, mitochondria and various mitochondria-associated proteins and myosin isoforms, to determine their distribution and arrangement at nanoscale in 3D in the fly. These studies could primarily utilize the rapid and inexpensive DiExM approach complemented with cryo-EM and X-ray and neutron scattering studies. Biochemical remodeling of the brain and skeletal muscle myosin and mitochondria could also be assessed using mass spectrometry. To assess functional changes to the neural circuits in the fly brain, genetically targeted optical electrophysiology could be performed. In the same way that genetically encoded fluorescent sensors revolutionized the study of intracellular calcium signals, ArcLight technology^29^ now enables optical measurements in intact neural circuits of membrane potential that underlies neuronal information processing and could be used to assess functional remodeling of neural circuits in the fly brain in exercised and sedentary states. Using this approach, multiple fluorescent voltage indicators could be genetically targeted and expressed in neurons of the fly brain, enabling reliable recording of individual electrical events simultaneously in multiple neurons according to published procedures^29^.

### 2.3 Focus 3: Computational machine learning approach to capture key elements in brain-muscle cross-talk, function, and regulation

To understand how the brain and skeletal muscles metabolism are interconnected, machine learning will be used to capture key elements in brain-muscle crosstalk.

#### (3a) The skeletal muscle component

Skeletal muscles account for more than a third of our body weight, and myocytes metabolism has a major impact on whole-body homeostasis. For instance, myocytes are responsible for ~75% of the insulin-stimulated clearance of glucose from the blood after a meal^30^. Mitochondria are key determinants of muscle cell functions controlling the spatiotemporal supply of energy where/when needed with dynamics (i.e. fusion vs fission), biogenesis, mitophagy and to-the-nucleus retrograde signaling shaping the mitochondrial intracellular content, architecture and interaction with other subcellular components. Moreover, all these mitochondrial hallmarks interplay with the metabolic state of the cell and the actual availability of respiratory substrates. In this context, using human myocytes differentiated into myotubes it is possible to assess accurately: (i) mitochondrial respiratory activity by high resolution oximetry and fluxes of major reporter metabolites for glycolysis, pentose shunt, lipid biosynthesis, mitochondrial import, TCA cycle, Branched Chain Amino Acid and one-carbon metabolism by SeaHorse technology or traditional biochemical/spectroscopic assays; (ii) mitochondrial morpho-functional properties by confocal microscopy imaging using specific fluorescent probes to detect morphology, mitochondrial membrane potential (mtΔΨ), reactive oxygen/nitrogen species, mtCa^2+^; (iii) expression of transcription factors and down-stream targets involved in mitochondrial biogenesis, mitophagy, dynamics, antioxidant response, and mitochondrial Unfolded Protein Response (mtUPR). Furthermore, as cellular molecular motors (myosin) consume energy in the form of ATP and convert it into mechanical work to generate force and movement, the heat loss by such a motor while performing work provides a way of quantifying its efficiency. Hence when equal amount of energy is used by a molecular motor, greater heat loss reflects lower efficiency. Based on this principle, a nanothermometry (NT) approach can be used to make precise measurements of mitochondria and myosin efficiency. Ultimately, regardless of how accurate and thorough the biochemical analysis might be, it is necessarily restricted to a limited number of parameters and thus is not able to cover all metabolic pathways of the cells under study. Fortunately, using a small number of experimentally derived parameters as constraints, the complexity of human cells metabolism can be explored computationally through state-of-the-art genome-scale metabolic models (GEMs)^31-33^. GEMs consist of a set of mass-balanced metabolic reactions connected into a network of shared metabolites, and suitable for Flux Balance Analysis (FBA). Starting from the generic metabolic network of a human cell, reconstruction of a context-specific GEM that capture the active subset of metabolism present in a particular tissues and cell types requires determining, for each metabolic reaction, whether or not it should be present, while simultaneously maintaining a functioning model^34,35^.The evidence for the presence of a reaction can be determined experimentally by deriving the existence of the relevant metabolites and enzymes from high-quality data with genome-wide coverage. A comprehensive myocyte GEM, iMyocyte2419, was recently reconstructed by^36^ using muscle precursor cells isolated from the human vastus lateralis muscle and differentiated into myotubes and can be downloaded in the systems biology markup language (SBML) format from the repository of curated models at http://www.metabolicatlas.org. It consists of 5,590 reactions in eight different compartments, 4,448 metabolites (2,396 unique), and 2,419 genes. The myotubes used to derive iMyocyte2419 were shown to be able to recapitulate known phenotypes of their donors, including known differences between males and females, such as higher lipid and energy metabolism in females, and metabolic anomalies associated with Type 2 diabetes. For this reason, iMyocyte2419 GEM could be adopted as the basic GEM for muscle metabolism, with updates/corrections based on locally acquired proteomic and transcriptome data. The metabolic pattern of human muscle cells in culture is expected to vary considerably depending on the growth conditions and phase. For example:

**A.** During log-phase on 2D/3D substrate cells will maximize the biomass synthetic reaction.

**B.** During stationary phase on either 2D or 3D substrates cells will minimize the energy demand and therefore ATP synthesis.

**C.** During stretching exercise on a 3D platform, cells will maximize the synthesis of ATP to support contraction.

**D.** After prolonged exercise myosin synthesis and mitochondrial biogenesis will be induced.

The reorganization of metabolism in these states will be reconstructed using the iMyocyte2419 model within the theoretical frame of Flux Balance Analysis (FBA)^37,38^. In FBA the traditional representation of the vector of time derivatives of metabolites concentrations **x **as the product of a rate matrix **K** and **x** is replaced by a product of a stoichiometric matrix **S **and a vector **v** of fluxes:

dx/dt=Kx → dx/dt=S*v*

The *stoichiometric matrix* is organized such that every column corresponds to a reaction (**r**) and every row corresponds to a compound (**c**), and thus it contains information on all the chemical reactions taking place in a cell. The entries in the matrix are *stoichiometric coefficients*, which are integers. Every row describes the reactions in which that compound participates and therefore how the reactions are *connected* by it. Therefore, the *dynamic mass balance equation*:

dx/dt=S*v*

describes all possible *dynamic *states of the metabolic system, with the *flux balance equation*:

S*v*=0

representing all possible combinations of metabolic fluxes consistent with a *steady-state*. Flux Balance Analysis seeks to maximize or minimize an objective (cost) function:

Z=c^T^*v*

where **c** is a vector of weights indicating how much each reaction (such as the biomass reaction when simulating maximum growth) contributes to the objective function. In practice, when only one reaction is desired for maximization or minimization, ***c***is a vector of 0 with a value of 1 at the position of the reaction of interest. Optimization of such a system is accomplished by a class of algorithms known as *linear programming^38^.* FBA can thus be defined as the use of linear programming to identify the best cost function:

min/max c^T^*v* s.t. {S*v* = dx/dt=0; *v*_i,min_ ≤ *v*_i_ ≤ *v*_i,max_

Where *v*_i,min_ ≤ *v*_i_ ≤ *v*_i,max_ are upper and lower limits on particular fluxes of metabolites as determined experimentally (see above). The output of FBA is a particular flux distribution, *v*, which maximizes or minimizes the objective function. Thus, the most important step in the application of FBA is the choice of the *objective function* that allows the identification of a particular functional state in the space of possible solutions. The most interesting types of objective functions are those that represent physiological functions or bioengineering objectives. Some examples with direct application to the functional states of muscle cells:

*Minimize or maximize ATP production*: find conditions of optimal energy efficiency.

*Minimize (or maximize) nutrient uptake*: find conditions to carry out a particular function with minimal or maximal consumption of certain nutrients (i.e., when testing the performance of myocytes contraction using glucose, fatty acids, or ketone bodies as substrates). 

*Minimize the 1- or 2-norm of the flux vector*: determine how a cell, channels metabolites at the lowest overall flux. Since protein synthesis is expensive, it is worthwhile for the cell to find a way to keep functioning using the lowest amounts of enzymes in its metabolic pathways. 

*Maximize metabolite production*: find the maximal production rate of a chosen metabolite (i.e., the sets of amino acids involved in the biosynthesis of myosin and/or mitochondrial proteins).

*Maximize biomass formation*: determine the maximal growth rate of a cell in a given environment. For example, this metabolic pattern would reflect the replication of muscle stem cells after an injury leading to muscle loss.

*Maximize biomass and metabolite production*: find the best compromise between cell growth and metabolite production. For example, myocytes propagated in a new culture after extensive exercise on a 3D stretching platform will both reenter log-phase (biomass maximization) and show evidence of exercise adaptation (increased myosin synthesis).

Additional applications of FBA are based on *sensitivity analysis*: in these studies, the metabolic changes that occur in muscle cells are derived when the flux through one reaction is varied and the optimal objective value is calculated as a function of this flux. Many combinations of reaction and objective can be investigated. For example, myocytes contraction can occur under aerobic or anaerobic conditions, and it is of interest to calculate the effect of varying glucose uptake, first by setting the oxygen uptake rate to the value observed experimentally by oxymetry (see above), and then by setting it to 0 (matching experiments in which our 3D platform is transferred to an anaerobic glove box). Finally, based on proteomic and transcriptome data, gene expression effects (including those produced by specific knock-outs) on metabolism can be investigated by constraining the associated reaction or reactions to specific values.

#### (3b) The brain component

The brain is composed of several cell types, including neurons and astrocytes. Highly optimized GEM’s are available for the cerebral cortex, hippocampus, and lateral ventricles neurons and glia cells, and for cerebellum granular and molecular layer cells and for Purkinje cells (http://www.metabolicatlas.org). These GEM’s allowed highly refined simulations of metabolic fluxes in these individual cell types. However, as result of the intimate connections between these cells, brain metabolism has been mostly modeled by multi-cell-type metabolic networks^39,40^. For example, a small-scale brain network of astrocyte and neuron cells was used to depict the effects of cerebral hypoxia on fluxes of different reactions^41^. In a different study, a small-size brain network was used to investigate the biochemical pathways involved in brain energetics^42^. In this case, random sampling of the flux space was used together with a modified version of FBA, called statistical flux balance analysis, in order to test several hypotheses about fuel supply of brain metabolism. For example, it was observed that the oxidative phosphorylation in a neuron is not sensitive to glucose uptake. More recently, a medium-scale two-compartment brain metabolic model has been reconstructed, which includes 570 genes and 630 metabolic reactions in astrocytes and neurons and the exchange reactions between these two compartments^43^. Using transcriptome data and gene-protein-reaction relations of the model, the authors evaluated the metabolic states of six neurodegenerative diseases with the aim of elucidating common and specific metabolic features of the diseases. Astrocytes, 3 types of neurons, plus a blood compartment and an interstitial space formed the in silico metabolic model of the brain organ. Flux constraints suitable for modeling neurons and glia metabolism will be derived (*in vitro*) from cell cultures biochemical studies (e.g., by SeaHorse technology) and *in vivo* from PET imaging. PET scans with ^15^O-O_2_, ^15^O-H_2_O, ^18^F-FDG, co-registered to T1-weighted magnetic resonance (MR) images, could be used to calculate Cerebral metabolic rate of oxygen (CMR_O__2_) and glucose (CMR_glu_) consumption, which are sufficient to constrain the FBA models. However, one limitation of the metabolic information derived from PET imaging is its modest time resolution, with observable changes occurring over a time scale of several minutes at best. This problem could be overcome by combining functional brain imaging with magnetoencephalography (MEG)^44^. The complex analysis procedure for MEG signals consists of various stages including co-registration with MRI/PET images, forward and inverse problem as well as applicable steps for data analysis. Subjects participating in such a MEG study will therefore also undergo MRI/PET for 3D anatomical images. Raw MEG data could be acquired from the subjects in a magnetically shielded room either in resting condition (no task) or during certain tasks (i.e. an exercise activity for a given muscle or group of muscles). Co-registration of MEG data with anatomical 3D MRI and PET images will enable source localization of the MEG data to specific brain regions. Ultimately, the combination of PET and MEG imaging with FBA metabolic reconstructions will lead to the development of a *metabolic map* of brain and skeletal muscles with millisecond and voxel scale resolution, and thus reveal the reciprocal influence and regulation of these two organs.

## 3. Conclusion

Metabolism and movement are among the critical determinants in the survival and success of an organism and are tightly regulated by the brain and skeletal muscle. At the cellular level, mitochondria -that powers life, and myosin - the cell’s molecular motor, have both evolved to serve this purpose. The design and approach discussed in this article utilizes a multi-disciplinary team of experts^45^ with new and novel tools and expertise in muscle physiology, neurobiology, computer engineering and machine learning, to establish a dedicated knowledge and technology base to progress our understanding in metabolism and movement. The approach outlined focuses on studies to understand how the brain and skeletal muscles communicate to regulate metabolism. How do brain and skeletal muscles adapt to inputs from each other? How does exercise impact the neural network of the brain, and how machine learning can be used to capture key elements in brain-muscle cross-talk in understanding metabolism and movement? Such a research program will additionally contribute to the technological advancements in tool-building and in new and novel approaches required for the proposed study and their application in chemistry, physics, engineering, and the biological sciences. In addition to contributing to the knowledge and technology base on metabolism and movement, and their application, the proposed program will have a transforming impact on society by educating the public on critical determinants of life, and utilization of the new and leading technologies. The science and technology base to be provided by the proposed program will serve as a resource to build on programmatic strengths, for the development of new technologies and their use and commercialization, for the transfer of knowledge to the broader scientific community and to public and private sectors, in the development of new courses and education programs for graduate and postdoctoral fellows, and for the recruitment of new faculty. The advanced tools and technologies created by the program, will be optimized and delivered for broader application in the investigation of a wide range of biological processes. The new approaches and ideas developed will also provide an unprecedented understanding of life processes. The proposed program will serve as a pipeline of trained scientists in the field who from the very beginning, are trained to engage the public. This highly interdisciplinary program with a futuristic vision, will serve the rapidly growing application of science and technology in service to humanity.
